# Flavonoids of Mao Jian Green Tea Ameliorate Glycemic Metabolism in Type-2-Diabetic Rats via AMPK Signaling Pathways and Gut Microbiota Regulation

**DOI:** 10.3390/foods14132402

**Published:** 2025-07-07

**Authors:** Lei Wu, Yao Niu, Fei Liu, Jiongling Tian, Zhilin Ma, Jiahui Yang, Xiaomeng Guo, Yaogui Sun

**Affiliations:** 1 College of Life Sciences, Shanxi Agricultural University, Taigu, Jinzhong 030801, China; n248199@163.com (Y.N.); lf02927@163.com (F.L.); sofiachenxz@outlook.com (J.T.); mzl1917242@163.com (Z.M.); yjh8071@163.com (J.Y.); gxm2092120@163.com (X.G.); 2 College of Veterinary Medicine, Shanxi Agricultural University, Taigu, Jinzhong 030801, China

**Keywords:** hepatic glycogen synthesis, insulin resistance, glucose-6-phosphatase, phosphoenolpyruvate carboxykinase, *Akkermansia muciniphila*

## Abstract

Mao Jian Green Tea flavonoids (MJGT_F) contain luteolin, luteolin-7-O-glucoside, eriodictyol, and eriodictyol-7-O-glucoside, among which the first three components have hypoglycemic effects; however, the overall hypoglycemic potential of MJGT_F remains unclear. This study demonstrated that MJGT_F inhibited α-glucosidase in vitro and improved metabolic parameters in a dose-dependent manner in T2DM (type 2 diabetes mellitus) rats (reducing blood glucose, triglyceride, total cholesterol, low-density lipoprotein, insulin, and the homeostatic model assessment of insulin resistance; increasing high-density lipoprotein, insulin sensitivity index, and glucagon-like peptide-1). High-dose MJGT_F (MJGT_F_H) showed optimal efficacy. Mechanistically, MJGT_F_H activated the AMPK pathway, evidenced by a significant increase in the p-AMPK/AMPK ratio and downregulation of hepatic gluconeogenic enzymes G6Pase and PEPCK. These coordinated effects collectively suggest enhanced hepatic glucose utilization and suppression of glucose overproduction. MJGT_F_H also modulated gut microbiota by enriching beneficial taxa (e.g., *Akkermansia muciniphila*, 11.17-fold vs. metformin) and reducing pathogens like Enterobacteriaceae. These findings highlight MJGT_F’s dual regulatory roles in glucose metabolism and microbiota, supporting its potential for diabetes management.

## 1. Introduction

Diabetes mellitus is a chronic endocrine metabolic disorder resulting from the combined influence of genetic predisposition and environmental factors. It is characterized by absolute or relative insulin deficiency and/or pancreatic beta-cell dysfunction, leading to dysregulated glucose and lipid metabolism in the body. The primary clinical manifestations include weight loss, polyphagia (excessive eating), polydipsia (excessive thirst), and polyuria (excessive urination). In 2021, the International Diabetes Federation (IDF) estimated that 537 million people worldwide had diabetes, representing 10.5% of the global population, with projections indicating that this number could exceed 640 million by 2045 [1]. The key drivers of this epidemic include the rising global burden of obesity, sedentary lifestyles, unhealthy diets, and population aging [2].

It is well-established that diabetes primarily comprises four common types: (1) type 1 diabetes mellitus (T1DM), characterized by T-cell-mediated destruction of beta cells leading to absolute insulin deficiency; (2) type 2 diabetes mellitus (T2DM), which manifests when pancreatic beta-cell insulin secretion becomes defective and insulin-sensitive tissues fail to respond adequately to insulin; (3) gestational diabetes mellitus (GDM), presenting as impaired glucose tolerance during pregnancy, a state where the mother requires increased nutrient absorption to support fetal development; and (4) monogenic diabetes, caused by mutations in a single gene or gene cluster [3].

T2DM, the most prevalent form of diabetes, accounts for over 90% of all diabetes cases [4]. Beyond hyperglycemia, its clinical manifestations encompass adipokine dysregulation, chronic inflammation, glucagon biology abnormalities [1], increased renal glucose reabsorption, and gut microbiota disturbances [2]. However, the core pathological mechanisms primarily involve pancreatic β-cell dysfunction and insulin resistance (IR) [4], driving therapeutic strategies focused on enhancing insulin secretion and improving insulin sensitivity. Current mainstream treatments include oral hypoglycemic agents and insulin injection therapy. Oral hypoglycemic medicines are categorized into multiple classes: insulin secretagogues (e.g., sulfonylureas), biguanides (e.g., metformin), insulin sensitizers (e.g., thiazolidinediones), α-glucosidase inhibitors (e.g., acarbose), incretin mimetics (e.g., GLP-1 receptor agonists), and SGLT-2 inhibitors [5]. Nevertheless, certain medications exhibit adverse effects. For instance, insulin secretagogues may induce hypoglycemia [6], biguanides can provoke gastrointestinal distress [7], and thiazolidinediones may elevate the risk of heart failure [8]. Insulin therapy remains irreplaceable in insulin-deficient diabetes (e.g., T1DM and advanced T2DM). Exogenous insulin injections are critical for sustaining patient survival but may trigger adverse reactions such as hypokalemia (due to insulin-driven intracellular potassium influx) and allergic responses. Furthermore, insulin co-administration with other medications (e.g., pioglitazone) may synergistically increase heart failure risk [9]. Consequently, the development of safer and more effective insulin replacement technologies or adjunctive therapies remains a pivotal research priority in diabetes management.

Over the past decade, the global adoption of complementary and alternative medicine (CAM) for managing chronic diseases such as diabetes has surged. Epidemiological surveys have revealed that up to 72.8% of diabetic patients utilize herbal medicines, dietary supplements, or other CAM modalities [10]. This trend aligns with the increasing recognition of medicinal plants for diabetes management. According to the World Health Organization (WHO), approximately 1200 medicinal plants worldwide exhibit significant antidiabetic properties, offering preventive or alternative therapeutic options for T2DM [11]. Commonly employed herbs and supplements include *Momordica charantia*, *Trigonella foenum-graecum*, *Gymnema sylvestre*, *Azadirachta indica*, L-carnitine, vanadium, chromium, and vitamin E. Their hypoglycemic mechanisms encompass diverse pathways: direct stimulation of insulin secretion, activation of hepatic glycogen synthesis and glycolysis, adrenomimetic effects, blockade of pancreatic β-cell potassium channels, cyclic adenosine monophosphate (cAMP) activation, and modulation of intestinal glucose absorption [12].

Flavonoids, a ubiquitous class of plant secondary metabolites, have demonstrated remarkable hypoglycemic activity and potential for mitigating diabetic complications [13]. A dose-response analysis revealed a 19% reduction in T2DM risk among individuals with the highest flavonoid intake (1202 mg/d) compared to those with the lowest intake (174 mg/d). Notably, flavonol, flavanol, and anthocyanin subclasses exhibit significant inverse correlations with T2DM incidence [14].

Mao Jian Tea, a traditional herbal tea from the Northern region of Shanxi Province, China, is derived from *Dracocephalum rupestre* Hance. For centuries, local communities have consumed this tea to alleviate postprandial abdominal distension associated with highland crops like naked oat and buckwheat. Based on processing methods, it is categorized into non-fermented Mao Jian Green Tea (MJGT) and fermented Mao Jian Black Tea (MJBT). Our prior research identified eriodictyol, eriodictyol-7-O-glucoside, luteolin, and luteolin-7-O-glucoside as major bioactive constituents in MJGT hydro extract (MJGT_HE), which demonstrated gastrointestinal motility regulation and gut microbiota modulation [15]. Intriguingly, these compounds exhibit hypoglycemic potential: Eriodictyol enhances glucose uptake and ameliorates insulin resistance via PI3K/AKT-mediated AKT phosphorylation [16] and stimulates insulin secretion through the cAMP/PKA pathway [17]. Luteolin and its glycoside derivative significantly improve glycemic parameters (fasting glucose, HbA1c, insulin levels, and homeostatic model assessment of insulin resistance) in diabetic KKAy mice, with luteolin outperforming its glucosylated form [18].

Based on these findings, we hypothesize that MJGT flavonoids possess untapped antidiabetic potential. To validate this, we first optimized a flavonoid-enriched extraction protocol to obtain MJGT flavonoids (MJGT_F). Subsequent in vivo experiments evaluated its hypoglycemic efficacy and mechanisms in a T2DM rat model. This study provides critical evidence for developing MJGT-based interventions and advancing its application in diabetes management.

## 2. Materials and Methods

### 2.1. Extraction of MJGT

MJGT was purchased from Jiufeng Cooperative (Ningwu, Shanxi, China) and authenticated by Professor Liwei Zhang from Shanxi University.

MJGT_HE: MJGT (17 g) was weighed and extracted with distilled water, and it continued to be heated for 20 min (stirring with a glass rod after boiling) to obtain MJGT_HE at a final concentration of 17 mg/mL.

MJGT_F: MJGT (200 g) was weighed out, 70% ethanol was used at a solid-to-liquid ratio of 1:20 (g/mL), it was heated under reflux for 60 min to extract, and 81 g of fluid extract was obtained. The MJGT alcoholic extract was further purified by polyamide column chromatography using a water–ethanol gradient elution system. Specifically, the column was sequentially eluted with water (3 bed volumes, BV) and then 10% ethanol (3 BV), followed by 15 BV of 80% ethanol at a flow rate of 1 mL/min. The obtained fractions were concentrated using a rotary evaporator (200 mbar, 45 °C), dried in an oven (50 °C), and weighed to yield 32.2 g of the extract.

### 2.2. Identification of Major Chemical Components in MJGT_F

Test sample preparation (S1): A stock solution of MJGT_F was prepared by accurately weighing 5.00 mg of the extract and dissolving it in 5 mL methanol to achieve a final concentration of 1 mg/mL. The solution was filtered through a 0.22 μm membrane to remove insoluble particulates.

Standard solution preparation (S2): Reference standards, including eriodictyol-7-O-glucoside (1.74 mg, DS0236, Chengdu DeSiTe Biological Technology Co., Ltd., Chengdu, China), eriodictyol (1.00 mg, DS0042, Chengdu DeSiTe Biological Technology Co., Ltd., China), luteolin-7-O-glucoside (1.35 mg, DM0016, Chengdu DeSiTe Biological Technology Co., Ltd., Chengdu, China), and luteolin (0.78 mg, DM0032, Chengdu DeSiTe Biological Technology Co., Ltd., Chengdu, China), were individually prepared in 5 mL volumetric flasks by dissolving each compound in HPLC-grade methanol (Thermo Fisher Scientific Inc., Waltham, MA, USA) to achieve final concentrations of 0.348, 0.206, 0.270, and 0.156 mg/mL, respectively. Calibration curves were constructed by serial dilution (10, 50, 200, 800, and 1000 μL aliquots adjusted to 1 mL with methanol).

HPLC conditions: The test solution and the standard solutions were loaded on an Agilent 1200 liquid chromatography system (Agilent Technologies, Inc., Santa Clara, CA, USA) equipped with a quaternary solvent delivery system, an autosampler, and a DAD detector, for determining the corresponding compounds and their contents. The separation was carried out on an Agilent TC-C18 column (250 mm × 4.6 mm, 5 μm) (Agilent 1260 II, Agilent Technologies, Inc., Santa Clara, CA, USA) according to the method established in our laboratory. In brief, the analysis was carried out by gradient elution with a mobile phase consisting of solvent A (0.3% aqueous acetic acid, *v*/*v*, chromatographic grade, Tianjin Comio Chemical Reagent Co., Ltd., Tianjin, China) and solvent B (HPLC-grade methanol Thermo Fisher Scientific Co., Ltd., Beijing, China). The gradient elution was set as follows: 32% B from 0 to 22 min, 32~37% from 22 to 23 min, 37% from 23 to 36 min, 37~45% B from 36 to 37 min, 45% B from 37 to 46 min, 45%~60% B from 46 to 47 min, 60~80% B from 47 to 60 min. UV absorption of eriodictyol and eriodicty-7-O-glucoside was monitored at 284 nm while luteolin and luteolin-7-O-glucoside was 350 nm. The column temperature was set at 25 °C. The flow rate was 1.0 mL/min, and the sample injection volume was 10 μL [15].

### 2.3. α-Glucosidase Activity Assay

The α-glucosidase inhibitory activity was evaluated following a modified protocol adapted from Tan et al. [19]. Briefly, 20 μL of PBS (pH 7.0, Catalog#AR0032, Boster Biological Technology, Wuhan, China) and 25 μL of α-glucosidase solution (1 U/mL, A875218, Shanghai Macklin Biochemical Co., Ltd., Shanghai, China. A875218) were added to a 96-well plate. Subsequently, 25 μL of MJGT_HE or MJGT_F at varying concentrations was introduced, followed by incubation at 37 °C for 15 min. The reaction was initiated by adding 100 μL of p-nitrophenyl-α-D-glucopyranoside (PNPG, N814494, Shanghai Macklin Biochemical Co., Ltd., China) and further incubated at 37 °C for 15 min. The enzymatic reaction was terminated with 80 μL of 0.1 M Na_2_CO_3_ solution. Acarbose (Product No. A9281, Beijing Solarbio Science & Technology Co., Ltd., Beijing, China) solution at a concentration range of (12.5–1000 μg/mL) was used as the positive control for the inhibition assay. Absorbance was measured at 405 nm using a microplate reader, with blank and background controls included. The inhibition rate was calculated as described below, and all enzymatic activity tests were performed in triplicate [20].

$$\alpha -\mathrm{Glucosidase}\;\mathrm{Inhibition}\;\mathrm{Rate}=\left(1\;-\;\frac{A_{\mathrm{Sample}\;}-{\;A}_{\mathrm{blank}}}{A_{\mathrm{test}}\;-\;{\;A}_{\mathrm{control}}}\right)\times \;100\%$$

*A_sample_*: Reaction system containing the test sample, α-glucosidase, and PNPG; *A_blank_*: Mixture of the sample (without enzyme) and PNPG. *A_test_*: Mixture of α-glucosidase and PNPG without the test sample. *A_control_*: The PNPG system with the enzyme but without the sample.

### 2.4. Establishment of T2DM Rat Model

Forty-six male Sprague-Dawley (SD) rats (180–200 g) were housed under standard laboratory conditions at Shanxi Agricultural University. All experimental protocols were approved by the Institutional Animal Ethics Committee of Shanxi Agricultural University (Approval No.: SXAU-EAW-2023SK.RP.012011194) and conducted in compliance with institutional guidelines.

The T2DM model was established based on a modified protocol from Jiao et al. [21]. Six rats were randomly assigned to the Negative Control (NC) group and fed standard maintenance chow, while the remaining rats received a high-fat high-sucrose (HFHS) diet for 4 weeks. The Model Group (MG) continued the HFHS diet throughout the experimental period. Subsequently, all groups except NC received an intraperitoneal (i.p.) injection of streptozotocin (STZ, 30 mg/kg in 0.1 mol/L citrate buffer, pH 4.5, S8050, Beijing Solarbio Science & Technology Co., Ltd, Beijing, China.) [22]. NC rats were administered an equivalent volume of citrate buffer (pH 4.5, 0.1 mol/L) via i.p. injection. All animals were fasted for 2 h post-injection to enhance STZ efficacy. Three days post-STZ administration, fasting blood glucose (FBG) levels were measured via tail-tip sampling after an overnight fast (ad libitum water access). Rats with FBG ≥ 11.1 mmol/L continued HFHS feeding for an additional week. Non-responsive animals (FBG < 11.1 mmol/L) received a second STZ injection (30 mg/kg, i.p.). After 3 d, FBG was re-evaluated, and rats persistently exhibiting FBG ≥ 11.1 mmol/L were classified as confirmed T2DM models.

### 2.5. Animal Grouping, Drug Administration, and Sample Collection

Excluding the NC group, 30 rats meeting glycemic criteria were randomly divided into 5 groups (6 rats per group): the MG, the Metformin Hydrochloride Group (MET, 250 mg/kg), and three flavonoid extract groups: the high-, middle-, and low-dose group of flavonoid extract from MJGT (MJGT_F_H, 136 mg/kg; MJGT_F_M, 68 mg/kg; MJGT_F_L, 34 mg/kg). All treated groups received daily oral gavage at 9:00 AM for 28 consecutive days. The NC and MG groups were administered an equivalent volume of distilled water. After the final administration, rats were fasted for 12 h (ad libitum water access), and then anesthetized. Blood was collected from the abdominal aorta and centrifuged at 2500 rpm for 10 min at −4 °C. Liver tissues were isolated from each group of euthanized rats, snap-frozen in liquid nitrogen, and stored individually at −80 °C for Western blot. Cecal contents were aliquoted into 1 mL sterile tubes, flash-frozen at −80 °C, and shipped on dry ice.

### 2.6. General Status Observation

During the administration period, we observed the food intake, water consumption, mental state, and activity levels of the rats. We recorded the body weight of the rats weekly.

### 2.7. Fasting Blood Glucose Measurement

We measured the FBG of the rats once weekly. The procedure was as follows: Prior to FBG measurement, each group of rats was fasted overnight (ad libitum water access). On the following morning, under calm conditions, blood glucose levels were measured from the tail tip vein of each rat.

### 2.8. Oral Glucose Tolerance Test

The oral glucose tolerance test (OGTT) is the most widely used clinical method for diagnosing glucose intolerance and diabetes [23]. After 26 d of drug administration, the rats are fasted for 12 h. On the following morning, 60 min after drug administration, a glucose solution (2.0 g/kg) is administered orally. Blood samples are collected from the tail vein at 0, 30, 60, 90, and 120 min to measure blood glucose levels. A blood glucose curve is plotted, and the area under the curve (AUC) is calculated to evaluate the OGTT of the rats.

### 2.9. Determination of Blood Lipid Parameters in T2DM Rats

Serum triglyceride (TG, order no. D799796), total cholesterol (TC, order no. D799800), and high-density lipoprotein cholesterol (HDL, order no. D799861) were measured using micro-methods, following the instructions provided in the respective kit manuals. Low-density lipoprotein cholesterol (LDL, order no. D731225), fasting serum insulin (InS, order no. D731159), and glucagon-like peptide-1 (GLP-1, order no. D731192) were measured using ELISA kits. All kits were purchased from Sangon Biotech (Shanghai) Co., Ltd., Shanghai, China. The following formulas were used to calculate Homeostatic Model Assessment of Insulin Resistance (Homa-IR) and the Insulin Sensitivity Index (ISI):

Homa-IR = (*FBG* × *InS*)/22.5

ISI = ln(*FBG* × *InS*) − 1

### 2.10. Western Blot

Liver tissues from the rats were weighed and ground under liquid nitrogen. The homogenized tissues were lysed in RIPA buffer (catalog no. C5029, Beijing Biosynthesis Biotechnology Co., Ltd., Beijing, China) containing 1% protease and phosphatase inhibitors, followed by incubation on ice for 30 min. After centrifugation (4 °C, 14,000 rpm, 10 min), the supernatant containing total protein was collected. Protein concentration was quantified using a BCA Protein Assay Kit (WLA004, Wanlei Life Sciences (Shenyang) Co., Ltd., Shenyang, China). Samples (10 μg per lane) were separated by sodium dodecyl sulfate–polyacrylamide gel electrophoresis (SDS-PAGE) and transferred to polyvinylidene fluoride (PVDF, IPVH00010, Merck KGaA, Darmstadt, Germany) membranes. After blocking with 5% non-fat milk powder for 1.5 h, antibodies (1:1000) were added against Glucose-6-phosphatase (G6Pase, bs-4044R, Beijing Biosynthesis Biotechnology, China), Phosphoenolpyruvate carboxykinase (PEPCK, D222686, Sangon Biotech (Shanghai) Co., Ltd., China), AMP-activated protein kinase (AMPK, bsm-33426M, Beijing Biosynthesis Biotechnology, Beijing, China), Phosphorylated AMP-activated protein kinase (p-AMPK, WL05103, Wanlei Life Sciences (Shenyang) Co., Ltd., Shenyang, China), and incubate at 4 °C for 12 h. After washing three times with TBST buffer (T1081, Beijing Solarbio Science & Technology Co., Ltd., China), the membranes were incubated with an HRP-conjugated Affinipure Goat Anti-Rabbit IgG(H+L) (WLA023, Wanlei Life Sciences (Shenyang) Co., Ltd., China) secondary antibody (1:5000) at room temperature for 1 h. Membranes were washed again three times with TBST, and protein bands were visualized using a ChemiDoc MP Imaging System (Bio-Rad Laboratories, Inc., Hercules, CA, USA). The expression levels of target proteins (G6Pase, PEPCK, AMPK, p-AMPK) were normalized to β-actin (used as the internal control protein) by calculating the ratio of target protein band intensity to that of β-actin for each sample.

### 2.11. 16S rDNA Sequencing

Microbial community analysis involved DNA extraction from rat fecal samples using the E.Z.N.A.^®^ Soil DNA Kit (Omega Bio-Tek, Inc., Norcross, GA, USA), followed by quality assessment via 1% agarose gel electrophoresis and quantification using a NanoDrop 2000 spectrophotometer (Thermo Fisher Scientific Inc., Waltham, MA, USA). The hypervariable V3-V4 regions of bacterial 16S rRNA genes were amplified via PCR (ABI GeneAmp^®^ 9700; Applied Biosystems, Thermo Fisher Scientific Inc., Waltham, MA, USA) employing universal primers 338F (5′-ACTCCTACGGGAGGCAGCAG-3′) and 806R (5′-GGACTACHVGGGTWTCTAAT-3′). Amplicons were purified using the AxyPrep DNA Gel Extraction Kit (Axygen Scientific, Inc., Union City, CA, USA) and quantified with a Quantus™ Fluorometer (Promega (Beijing) Biotech Co., Ltd., Beijing, China). Sequencing libraries were constructed with the NEXTFLEX Rapid DNA-Seq Kit (Bioo Scientific Corporation, Austin, TX, USA), and paired-end sequencing (2 × 300 bp) was performed on an Illumina MiSeq platform (Majorbio Bio-Pharm Technology Co., Shanghai, China). Raw sequences were processed using the UPARSE pipeline (v7.1) to cluster operational taxonomic units (OTUs) at 97% similarity. Taxonomic assignment was subsequently performed against the SILVA 16S rRNA database (v132) using the RDP classifier with a 70% confidence threshold.

### 2.12. Data Analysis

Data are expressed as mean ± SEM and statistically analyzed using one-way analysis of variance (ANOVA) with SPSS version 26.0 (SPSS, Inc., Chicago, IL, USA). Visualization was performed using GraphPad Prism version 10.1.2 (GraphPad Software, Inc., La Jolla, CA, USA).

## 3. Results

### 3.1. Identification of the Main Chemical Components of MJGT_F

**Figure 1:**
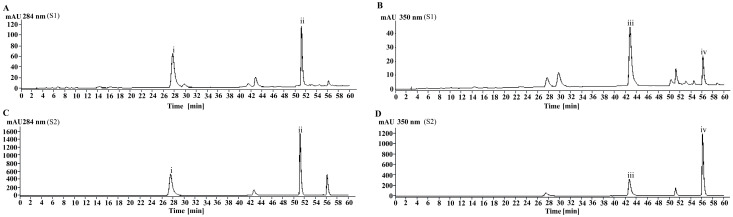
The chromatogram of the MJGT_F. (**A**) Analysis results of MJGT_F sample at 284 nm; (**B**) Analysis results of MJGT_F sample at 350 nm; (**C**) Analysis results of the four-standard mixture at 284 nm; (**D**) Analysis results of the four-standard mixture at 350 nm.

In this study, four main chemical components, namely eriodictyol-7-O-glucoside (i, 17.83 ± 0.06%), luteolin-7-O-glucoside (ii, 4.34 ± 0.04%), eriodictyol (iii, 14.73 ± 0.07%), and luteolin (iv, 6.00 ± 0.07%), were identified from MJGT_F using the method previously established in our laboratory [15] (Table S1 and Figure 1).

### 3.2. Inhibitory Effects of MJGT_F on α-Glucosidase Activity

**Figure 2:**
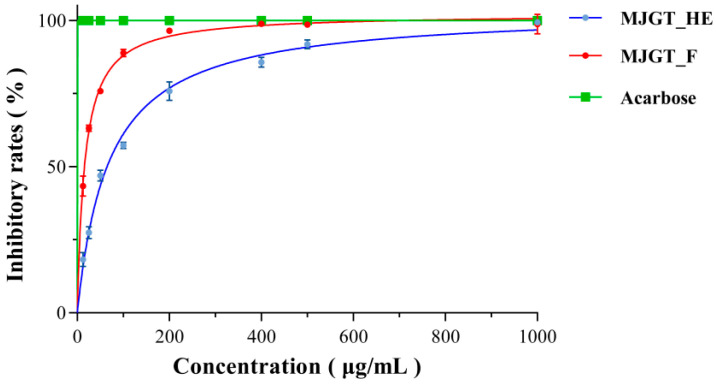
The inhibitory activity of MJGT_F and MJGT_HE against α-Glucosidase.

α-Glucosidase plays a pivotal role in intestinal glucose absorption [24]. The pharmacological inhibition of this enzyme represents an effective therapeutic strategy for mitigating postprandial hyperglycemia in T2DM. To elucidate the hypoglycemic mechanisms of MJGT_HE and MJGT_F, we systematically evaluated their inhibitory effects on α-glucosidase activity. Dose-response analyses revealed concentration-dependent inhibition patterns for both extracts, with MJGT_F demonstrating significantly stronger inhibitory potency than MJGT_HE at equivalent concentrations (Figure 2). Notably, the positive control acarbose (a clinical α-glucosidase inhibitor) achieved complete enzyme inhibition at concentrations ≤ 1000 μg/mL.

### 3.3. Effects of MJGT_F on Blood Glucose and Related Parameters in T2DM Rats

**Figure 3:**
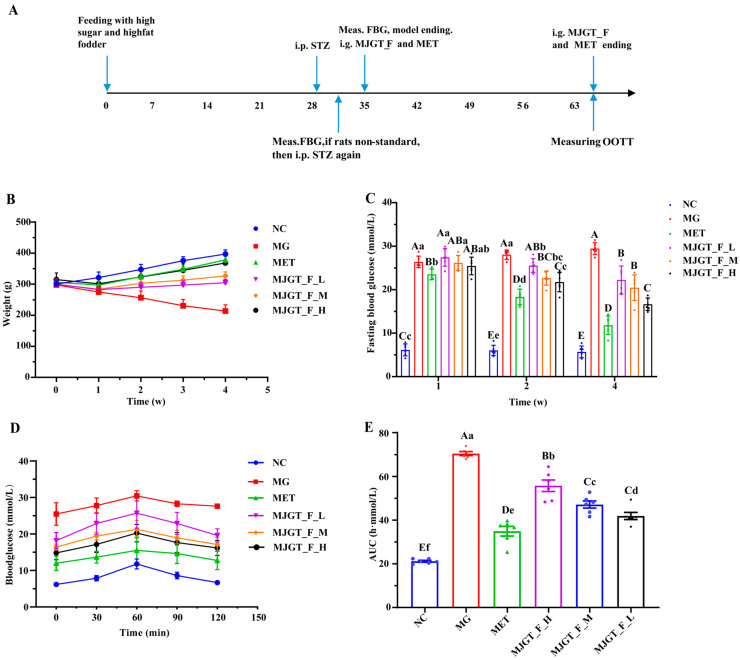
The timeline from rat model establishment and administration to sampling (**A**). Comparison of weight (**B**), fasting blood glucose (**C**), blood glucose (**D**), and AUC (**E**) in rats of different groups. Capital and lowercase letters above the bar indicate the difference significance at the 0.01 or 0.05 level, respectively. (*n* = 6 for each group). NC: Negative Control Group; MG: Model Group; MET: Metformin Hydrochloride Group; MJGT_F_H, MJGT_F_M, and MJGT_F_L represent the high-, middle-, and low-dose groups of flavonoid extract from MJGT, respectively.

A T2DM rat model was successfully established by feeding a high-fat, high-sugar diet for 28 d followed by STZ induction. The MG was then subjected to drug treatment, and relevant parameters were measured (Figure 3A). During the 4-week treatment period, the body weight of the MG continuously declined, with a reduction of 7.70 ± 1.51% in the first week and 28.26 ± 5.50% by the fourth week (Figure 3B). Although all treatment groups still exhibited varying degrees of weight loss in the first week (MET: 2.86 ± 0.88%; MJGT_F_L: 5.86 ± 1.66%; MJGT_F_M: 5.34 ± 1.21%; MJGT_F_H: 4.23 ± 0.84%), the MET showed a significantly slower decline. From the second week onward, all treatment groups began to regain weight, with their body weights significantly higher than that of the MG (256.58 ± 21.35) (*p* < 0.01). Notably, the MJGT_F_H and MET surpassed their initial weights by 5.90 ± 2.05% and 2.77 ± 1.21%, respectively. By the fourth week, the MET, MJGT_F_L, MJGT_F_M, and MJGT_F_H groups exhibited weight increases of 23.71 ± 4.58%, 1.51 ± 0.85%, 8.94 ± 1.93%, and 17.21 ± 3.91%, respectively, compared to baseline. Although the weight values of the MET group remained lower than those of the NC (397.17 ± 13.04), no significant difference was observed (*p* > 0.01) (Table S2).

In terms of fasting blood glucose (FBG), only the MET group demonstrated a significant hypoglycemic effect (*p* < 0.01) in the first week. By the second week, all treatment groups except MJGT_F_L showed notable reductions in FBG (*p* < 0.01). By the fourth week, all treatment groups exhibited significant decreases in FBG (*p* < 0.01), with reductions of 59.97 ± 6.35% (MET), 26.19 ± 7.95% (MJGT_F_L), 28.73 ± 8.77% (MJGT_F_M), and 43.5 ± 6.70% (MJGT_F_H) compared to the MG (Figure 3C).

During the OGTT, blood glucose levels peaked at 60 min in all groups before gradually declining (Figure 3D). While the NC and MET returned to baseline levels by 120 min, the MG maintained significantly elevated glucose levels (*p* < 0.01). The AUC analysis further confirmed that the MG had a significantly higher AUC than the NC (*p* < 0.01) (Figure 3E). Although all treatment groups exhibited lower AUC values than the MG group (*p* < 0.01), the MJGT_F treatments did not achieve the same efficacy as MET. Among the three MJGT_F doses, MJGT_F_H demonstrated the strongest hypoglycemic effect and was, thus, selected for subsequent experiments.

### 3.4. Effects of MJGT_F_H on Insulin and Related Parameters in T2DM Rats

**Figure 4:**
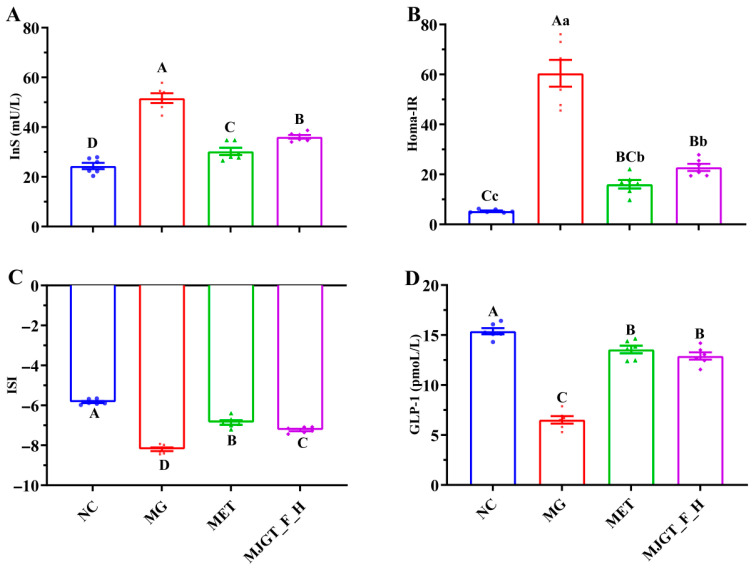
Effect of MJGT_F_H treatment on InS (**A**), HOMA-IR (**B**), ISI (**C**), and GLP-1 (**D**) in T2DM rats. Capital and lowercase letters above the bar indicate the difference significance at the 0.01 or 0.05 level, respectively. (*n* = 6 for each group). NC: Negative Control Group; MG: Model Group; MET: Metformin Hydrochloride Group; MJGT_F_H: the high-dose group of flavonoid extract from MJGT.

An evaluation of insulin-related parameters revealed significant elevations of InS and Homa-IR in the MG compared to NC (*p* < 0.01) (Figure 4A,B). After drug administration (MET or MJGT_F_H), the levels of the two parameters were significantly down-regulated (*p* < 0.01), but they failed to return to the levels of the NC. The changes in ISI levels (Figure 4C) mirrored those of GLP-1 (Figure 4D), showing significant downregulation in the MG. However, drug administration induced a marked elevation (*p* < 0.01), though levels remained below NC baseline values (Figure 4). Notably, while MJGT_F_H and MET demonstrated comparable efficacy in modulating Homa-IR and GLP-1 (*p* > 0.05), significant differences persisted in their effects on InS and ISI (*p* < 0.01).

### 3.5. Evaluation of MJGT_F_H on Serum-Related Indexes in T2DM Rats

**Figure 5:**
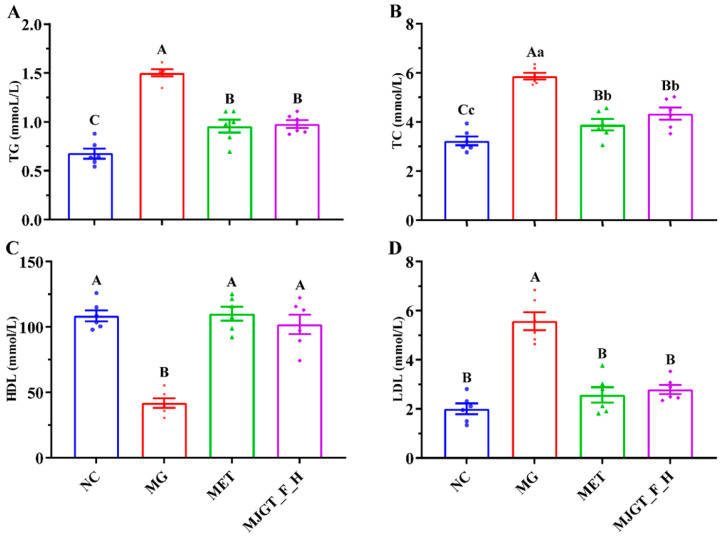
Effect of MJGT_F_H treatment on TG (**A**), TC (**B**), HDL, (**C**) and LDL (**D**) in T2DM rats. Capital and lowercase letters above the bar indicate the difference significance at the 0.01 or 0.05 level, respectively. (*n* = 6 for each group)**.** NC: Negative Control Group; MG: Model Group; MET: Metformin Hydrochloride Group; MJGT_F_H: the high-dose group of flavonoid extract from MJGT.

T2DM is often associated with disorders of glucose and lipid metabolism, so changes in lipid-related markers were also examined in this study. Compared with NC, three parameters, TG, TC, and LDL, were significantly increased (*p* < 0.01), and HDL was significantly down-regulated (*p* < 0.01) in MG, whereas both administration treatments restored the relevant indexes (*p* < 0.01), and there was no difference in the restorative capacity of the two. In particular, both of them were able to regulate LDL and HDL back to the NC level (Figure 5).

### 3.6. Modulation of AMPK Signaling Pathway by MJGT_F_H in T2DM Rats

**Figure 6:**
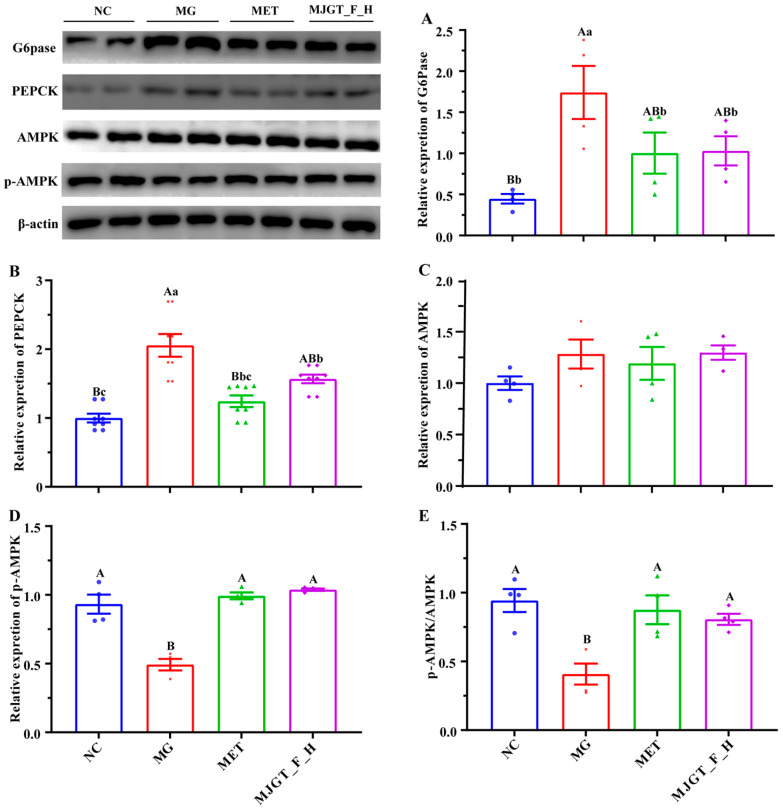
Effects of MJGT_F_H treatment on the expression of key genes and proteins in the AMPK signaling pathway in the liver of T2DM rats. (**A**) G6Pase, (**B**) PEPCK, (**C**) AMPK, (**D**) p-AMPK, (**E**) p-AMPK/AMPK. Capital and lowercase letters above the bar indicate the difference significance at the 0.01 or 0.05 level, respectively. (*n* = 4 for each group). NC: Negative Control Group; MG: Model Group; MET: Metformin Hydrochloride Group; MJGT_F_H: the high-dose group of flavonoid extract from MJGT.

To investigate the regulatory effects of MJGT_F_H on the AMPK pathway, we performed Western blot analysis to quantify key proteins involved in gluconeogenesis and energy metabolism, including G6Pase, PEPCK, AMPK, and p-AMPK. Comparative analysis revealed that the MG exhibited significantly upregulated expression of hepatic gluconeogenic enzymes G6Pase and PEPCK relative to NC (*p* < 0.01) (Figure 6A,B). While AMPK protein levels remained unchanged in MG, p-AMPK was markedly reduced (*p* < 0.05) (Figure 6C,D). Therapeutic intervention with either MET or MJGT_F_H maintained stable AMPK expression while effectively restoring p-AMPK levels to those observed in NC. Consequently, the p-AMPK/AMPK ratio showed parallel changes with p-AMPK expression patterns (Figure 6E).

### 3.7. Impact of MJGT_F_H on Gut Microbiota in T2DM Rats

Using 16S rDNA sequencing, we analyzed compositional changes in gut microbiota among different treatment groups. Based on the results of the principal coordinates analysis (PCoA), which reflects the similarity and difference levels of community composition among samples, it can be found in the figure that the horizontal axis (PC1) and the vertical axis (PC2) represent the first and second principal coordinates, respectively, with contribution rates of 18.41% and 16.88%, respectively. These two principal coordinates together account for 35.29% of the variation among the samples. NC and MG showed significant dispersion along both coordinates, indicating marked microbial differences. Both MET and MJGT_F_H treatments resulted in more clustered distributions, with MET partially overlapping both NC and MG, suggesting similar therapeutic effects that partially restored normal microbiota composition (Figure S1A).

Analysis at the phylum level revealed that the top five dominant bacterial phyla were Firmicutes, Bacteroidetes, Proteobacteria, Actinobacteria, and Verrucomicrobiota. Among them, Firmicutes and Bacteroidota had the highest proportions. In the NC, MG, MET, and MJGT_F_H groups, the proportions of Firmicutes and Bacteroidota were 60.40% and 13.11%; 85.73% and 8.09%; 78.97% and 10.62%; 62.63% and 20.33%, respectively (Figure S1B). The Firmicutes/Bacteroidetes (F/B) ratios were 4.61, 10.59, 7.44, and 3.08, respectively. These results indicate that the onset of diabetes can significantly alter the F/B ratio in rats (approximately 2.30-fold increase), and both drug administrations (MET and MJGT_F_H) can reverse this change, with MJGT_F_H treatment showing the best reversal effect.

At the family level, the model establishment had different effects on the abundances of different bacterial families, with some abundances increasing and others decreasing. The effects of the two drug treatments also showed two different trends. First, the abundances of some families decreased after model establishment but rebounded after drug treatment. For instance, the abundance of Akkermansiaceae changed the most before and after model establishment and drug treatment. Compared with the NC group (0.14%), the abundance in the MG group (0.0018%) decreased by 98.71%. After treatment with MET and MJGT_F_H, the abundances increased to 0.73% (405.55-fold increase) and 8.28% (4600-fold increase), respectively. The abundance of Eubacterium_coprostanoligenes_group decreased from 4.02% (NC) to 1.97% (MG) and increased to 4.45% (MET) and 4.67% (MJGT_F_H) after drug administration. After model establishment, the abundance of Muribaculaceae decreased from 12.03% in the NC group to 5.82%, and after drug administration, it rebounded to 9.32% (MET) and 12.31% (MJGT_F_H), respectively. The abundance of Enterobacteriaceae also changed significantly before and after model establishment, decreasing from 14.79% in the NC group to 1.66% (MG), a reduction of 88.78%. However, the two treatments also had quite different effects on its recovery. MET restored its abundance to 5.95%, while after MJGT_F_H treatment, its abundance was only 0.13%, showing a decrease. The proportions of Bifidobacteriaceae and Prevotellaceae in the MG group decreased by 70.65% (from 2.01% in the NC group to 0.59% in the MG group) and 86.76% (from 0.68% in the NC group to 0.09% in the MG group), respectively. Interestingly, after MET treatment, the abundance of the former continued to decrease to 0.28%, while the latter increased to 0.20%. MJGT_F_H could reverse the abundances of both, increasing them to 1.00% and 1.29%, which were 1.69-fold and 14.33-fold higher than those in the MG group, respectively. In contrast, the abundances of another part of families increased after model establishment and decreased after drug treatment. For instance, the abundance of Lactobacillaceae increased from 22.53% (NC) to 31.27% (MG) and then decreased to 21.03% (MET) and 14.50% (MJGT_F_H) after drug administration. The proportion of Peptostreptococcaceae increased from 4.10% in the NC to 9.66% in the MG, representing an approximately 1.36-fold increase. After drug administration, its abundance only slightly decreased to 9.10% in the MJGT_F_H group. Similarly, the abundance of Oscillospiraceae increased from 2.65% (NC) to 6.55% (MG) and then decreased to 6.12% (MET) and 5.93% (MJGT_F_H) after drug administration (Figure S1C).

At the species level, the abundance of *Akkermansia muciniphila* was 0.21% in the NC, decreased to 0.0021% in the MG, and showed a substantial rebound after drug treatment, reaching 1.01% (approximately 480.95-fold increase) and 11.28% (approximately 5371.43-fold increase) in the MET and MJGT_F_H groups, respectively. This is also the main reason for the significant change in the abundance of Akkermansiaceae. In the MG, compared with the NC, the proportions of *Lactobacillus johnsonii*, *Lactobacillus intestinalis*, *Lactobacillus reuteri*, and *Lactobacillus murinus* all increased to varying degrees, rising from 6.47%, 3.74%, 3.65%, and 2.19% to 10.16%, 10.03%, 8.77%, and 3.09%, respectively. After MJGT_F_H treatment, their proportions decreased to 2.68%, 2.01%, 1.63%, and 0.22%, respectively. After MET treatment, the proportions of the first three bacteria increased slightly to 8.72%, 4.35%, and 4.40%, respectively, while the proportion of *Lactobacillus murinus* decreased to 0.96%. The proportion of *Romboutsia ilealis* increased significantly in the MG (10.89%) compared with the NC (2.96%). After drug treatment, it decreased slightly, reaching 9.85% and 8.31% in the MET and MJGT_F_H, respectively. The proportion of *Escherichia coli_g__Escherichia-Shigella* decreased significantly after model establishment (0.07%) compared with the NC (22.22%). After administration of MET and MJGT_F_H, it rebounded to 4.11% and 0.19%, respectively (Figure S1D).

## 4. Discussion

This study aimed to investigate the therapeutic effects of flavonoid-rich MJGT_F_H on high-fat/high-sucrose diet combined with streptozotocin-induced T2DM and elucidate its underlying mechanisms. Obesity and high-fat diet promote activation of saturated fatty acid-stimulated adenine nucleotide translocase 2 (ANT2), an intramitochondrial protein that induces adipocyte hypoxia and subsequent hypoxia-inducible factor-1α (HIF-1α) activation, leading to adipose tissue dysfunction and inflammation [25,26]. Obese adipocytes further elevate pro-inflammatory cytokine levels, causing chronic low-grade systemic inflammation (metabolic inflammation) [26], a critical contributor to IR and T2DM pathogenesis [27]. Given the inverse correlation between flavonoid intake and body fat accumulation/weight gain [28], all three MJGT_F concentrations dose-dependently reversed T2DM-induced weight loss and suppressed both postprandial and fasting blood glucose levels, with MJGT_F_H showing the most potent effects. Mechanistic studies revealed that MJGT_F_H’s hypoglycemic effects operate through three interrelated pathways: metabolic regulation, signaling pathway modulation, and gut microbiota remodeling.

*Metabolic regulation* T2DM involves severe disturbances in glucose and lipid metabolism. MJGT_F_H significantly ameliorated dyslipidemia in diabetic rats, normalizing elevated TG, TC, and LDL levels while restoring reduced HDL levels. Crucially, it improved hyperinsulinemia by downregulating the HOMA-IR index and upregulating the ISI, indicating enhanced insulin sensitivity. MJGT_F_H also elevated serum GLP-1 levels, whose fluctuations hold significant physiological and pathological implications for diabetic patients. The underlying mechanisms primarily involve the following: binding to the GLP-1 receptor (GLP-1R) on pancreatic β-cells, which activates adenylyl cyclase (AC) to elevate intracellular cAMP levels, subsequently activating protein kinase A (PKA) and an exchange protein directly activated by cAMP 2 (EPAC2), thereby promoting insulin gene transcription and glucose-stimulated insulin secretion [29]; β-cell protection via apoptosis inhibition (e.g., reducing endoplasmic reticulum stress and mitochondrial dysfunction) and proliferation promotion (through Wnt/β-catenin and PDX1/MafA pathways), thereby improving long-term islet function [30]; suppression of glucagon secretion from pancreatic α-cells via the cAMP-PKA pathway, reducing hepatic glucose output [31]. MJGT_F_H likely restores pancreatic β-cell insulin secretion capacity by elevating serum GLP-1 levels in T2DM rats, thereby inducing β-cell proliferation and reducing apoptosis. Concurrently, this process enhances insulin utilization, mitigates IR, increases hepatic glycogen synthesis, and suppresses gluconeogenesis, collectively ameliorating T2DM-associated metabolic derangements.

*Signaling Pathway Modulation* The AMPK pathway plays a pivotal role in cellular metabolism and function, exerting profound effects on peripheral insulin action, glucose uptake, nutrient intake, lipid metabolism, inflammation, and insulin secretion. These comprehensive regulatory functions enable AMPK to critically influence systemic glucose and lipid homeostasis, rendering it an attractive therapeutic target for T2DM. Notably, reduced AMPK activity has been consistently observed in the skeletal muscle of both obese individuals and T2DM patients, as well as in corresponding animal models. This suppression may result from multiple mechanisms: hyperglycemia-induced disruption of the liver kinase B1 (LKB1) complex [32], activation of protein phosphatase PP2A [33], upregulation of PH domain and leucine-rich repeat protein phosphatase 2 (PHLPP2) [34], and ubiquitin-mediated degradation of AMPK subunits [35,36]. Our findings demonstrate that MJGT_F_H effectively activates the AMPK signaling cascade. Specifically, MJGT_F_H treatment significantly increased the ratio of p-AMPK to total AMPK while enhancing GLUT4 expression, thereby promoting GLUT4 translocation and subsequent cellular glucose uptake [37]. Concurrently, MJGT_F_H downregulated the expression of key gluconeogenic enzymes G6Pase and PEPCK, effectively suppressing hepatic glucose production [38]. This AMPK-mediated signaling modulation collectively improves hepatic insulin sensitivity, directly counteracts insulin IR, and corrects glucose metabolic disturbances in T2DM.

*Gut microbiota remodeling* The crucial role of the gut microbiota and its metabolites in the pathogenesis of diet-induced obesity and related metabolic disorders has been widely confirmed. Studies have shown that there are significant differences in the composition of bacterial phyla between T2DM patients and non-diabetic controls. In T2DM patients, the abundance of Firmicutes is significantly increased, resulting in a significantly higher F/B ratio compared to non-diabetic patients [39]. In this study, a 1.28-fold increase of F/B was observed in MG compared with the NC. After drug intervention, this ratio decreased significantly. In particular, the F/B ratio in the MJGT_F_H group decreased by 2.44 folds. The mechanisms by which MJGT_F_H improves T2DM symptoms through regulating the gut microbiota may involve the following aspects: (1) reducing the inflammatory response. Chronic inflammation is one of the characteristics of diabetes, which can lead to IR, impaired insulin secretion, and abnormal glucose tolerance. For instance, both Bifidobacteriaceae and Prevotellaceae have anti-inflammatory effects, but the anti-inflammatory mechanisms of these two in T2DM are not yet clear. In this study, the abundances of the above-mentioned families in the MJGT_F_H group were significantly reversed compared with the MG. Notably, the abundance of *Akkermansia muciniphila* exhibited the most dramatic alteration, showing a 5371.42-fold increase in the MJGT_F_H group compared to the MG group. Through its participation in luminal protection of the gastrointestinal tract, it reduces bacterial translocation, thereby improving fat storage, adipose tissue metabolism, and glucose homeostasis [40]. (2) Promoting the production of short-chain fatty acids (SCFAs). SCFAs are mainly produced by the fermentation of dietary fiber by gut microbiota, including acetic acid, butyric acid, and propionic acid. The SCFA levels in T2DM patients are generally lower than those in healthy people. Muribaculaceae produces SCFAs through endogenous (mucin glycans) and exogenous polysaccharides (dietary fiber) [41]. The intervention of MJGT_F_H significantly promoted the proliferation of this type of bacterial group. (3) Improving lipid metabolism. Due to the disorder of the biological regulatory function of insulin, T2DM is often accompanied by lipid metabolic disorders. The intervention of MJGT_F_H can promote the proliferation of the *Eubacterium coprostanoligenes* group, which helps to excrete excess cholesterol and other metabolic wastes, reduce the burden on the liver, and at the same time promote nutrient absorption and gut health by decomposing indigestible substances such as cellulose. In addition, *Akkermansia muciniphila* can down-regulate the levels of TG and Alanine aminotransferase (ALT) in serum by reducing the expression of the TG synthesis factor SREBP [42], thereby improving the metabolic abnormalities in obese mice. (4) Reducing the number of pathogenic bacteria. Enterobacteriaceae is prone to develop antibiotic resistance and may induce opportunistic infections in patients with low immune function. Notably, it was found that after the intervention of MJGT_F_H, the abundance of Enterobacteriaceae decreased by 50%, indicating its role in regulating the balance of the gut microbiota.

Although epidemiological studies have indicated a significant inverse correlation between the intake of flavonoids (including subclasses such as flavonols, flavanols, and anthocyanins) and T2DM risk, with a dose-threshold effect (risk significantly decreases at intakes ≥ 550 mg/day [43], or effects plateau beyond the 395–602 mg/day range [14]), this study did not thoroughly explore the specific associations between different flavonoid subclasses and glucose-lowering effects. While the results align closely with population data, confirming the definite glucose-lowering efficacy of flavonoids in the experimental intervention, only preliminary identification of the major active components in the extract was performed. There was a lack of systematic investigation into the bioavailability, tissue distribution, and metabolic transformation processes of key flavonoid monomers and their metabolites in vivo following administration. Subsequent studies should focus on the key active flavonoid monomers within MJGT_F_H, evaluating their individual and combined pharmacodynamic effects, and delving into their in vivo pharmacokinetic characteristics. This will significantly enhance the depth and targeting of mechanistic research.

## 5. Conclusions

The MJGT_F_H can ameliorate metabolic disorders in T2DM rats, and its mechanism of action primarily involves the following aspects: first, by upregulating the expression of p-AMPK in liver tissue, it activates the AMPK signaling pathway, thereby downregulating the expression of key gluconeogenic enzymes G6Pase and PEPCK. This enhances hepatic glycogen synthesis, suppresses hepatic gluconeogenesis, and alleviates IR. Second, by modulating the abundance of relevant gut microbiota (particularly the significant upregulation of *Akkermansia muciniphila*, which is directly involved in blood glucose regulation), it corrects abnormal glucose and lipid metabolism, thereby synergistically improving the pathological state of T2DM.

## Data Availability

The original contributions presented in the study are included in the article/Supplementary Materials. Further inquiries can be directed to the corresponding authors.

## Supplementary Materials

The following supporting information can be downloaded at https://www.mdpi.com/article/10.3390/foods14132402/s1. Table S1: Flavonoid content in the methanol-soluble fraction of MJGT_F. Table S2: Body weight changes in different groups during drug administration (*n* = 6). Figure S1: PCoA analysis of the OTU level of gut microbiota (A). The variations in gut microbiota composition are displayed the phylum (B), family (C), and species level (D), respectively. (*n* = 4 for each group). NC: Negative Control Group; MG: Model Group; MET: Metformin Hydrochloride Group; MJGT_F_H: high-dose group of flavonoid extract from MJGT.

## Abbreviations

The following abbreviations are used in this manuscript:

T2DM	type 2 diabetes mellitus
IR	insulin resistance
CAM	complementary and alternative medicine
WHO	World Health Organization
cAMP	cyclic adenosine monophosphate
MJGT	Mao Jian Green Tea
MJBT	Mao Jian Black Tea
MJGT_HE	MJGT hydro extract
SD	Sprague-Dawley
NC	Negative Control
HFHS	high-fat high-sucrose
MG	Model Group
STZ	streptozotocin
FBG	fasting blood glucose
MET	Metformin Hydrochloride Group
TG	serum triglycerid
TC	total cholesterol
HDL-C	high-density lipoprotein cholesterol
LDL-C	low-density lipoprotein cholesterol
Homa-IR	Homeostatic Model Assessment of Insulin Resistance
ISI	Insulin Sensitivity Index
InS	fasting serum insulin
SDS-PAGE	sodium dodecyl sulfate-polyacrylamide gel electrophoresis
PVDF	polyvinylidene fluoride
G6Pase	Glucose-6-phosphatase
PEPCK	Phosphoenolpyruvate carboxykinase
AMPK	AMP-activated protein kinase
p-AMPK	Phosphorylated AMP-activated protein kinase
PCoA	principal coordinates analysis
GLUT4	Glucose Transporter Type 4
SCFAs	short-chain fatty acids
ALT	Alanine aminotransferase
