# Supplementary material for: Flavonoids of Mao Jian Green Tea Ameliorate Glycemic Metabolism in Type-2-Diabetic Rats via AMPK Signaling Pathways and Gut Microbiota Regulation
Source: Foods. 2025 Jul 7;14(13):2402. doi: 10.3390/foods14132402 (PMC12249265; doi:10.3390/foods14132402)
Supplement: Supplementary file 1 [file foods-14-02402-s001.zip › foods-3684039-supplementary.pdf]

Table S1. Flavonoid content in the methanol-soluble fraction of MJGT_F

	flavonoids/mg	*concentration mg/ml*	*Content determination*			
			eriodictyol	*eriodictyol-7-O-glucoside*	*luteolin*	*luteolin-7-O-glucoside*
1	5.274	1.055	14.70 %	17.82 %	6.03 %	4.34 %
2	5.148	1.030	14.62 %	17.72 %	5.87 %	4.26 %
3	5.306	1.061	14.86 %	17.94 %	6.11 %	4.41 %
Mean±SEM	5.240±0.05	1.049±0.010	14.73±0.07 %	17.83±0.06 %	6.00±0.07 %	4.34±0.04 %

Table S2. Body weight changes in different groups during drug administration (*n*=6)

Group	***Time***				
	0 week	***1 week***	***2 weeks***	***3 weeks***	***4 weeks***
NC	300.50±13.32	321.42±17.6	347.75±15.88	376.83±13.53	397.17±13.04
MG	297.50±19.01	274.58±17.18	256.58±21.35	230.83±20.35	213.42±20.11
MET	306.17±11.17	297.42±11.80	324.08±8.40	347.92±8.58	378.50±12.35
MJGT_F_L	300.25±11.26	282.75±14.08	290.25±14.44	397.42±12.94	304.75±10.62
MJGT_F_M	299.92±12.31	284.00±14.31	302.83±11.69	313.00±13.18	326.67±12.85
MJGT_F_H	314.67±21.49	301.33±20.38	323.33±21.26	344.67±20.24	368.42±20.95

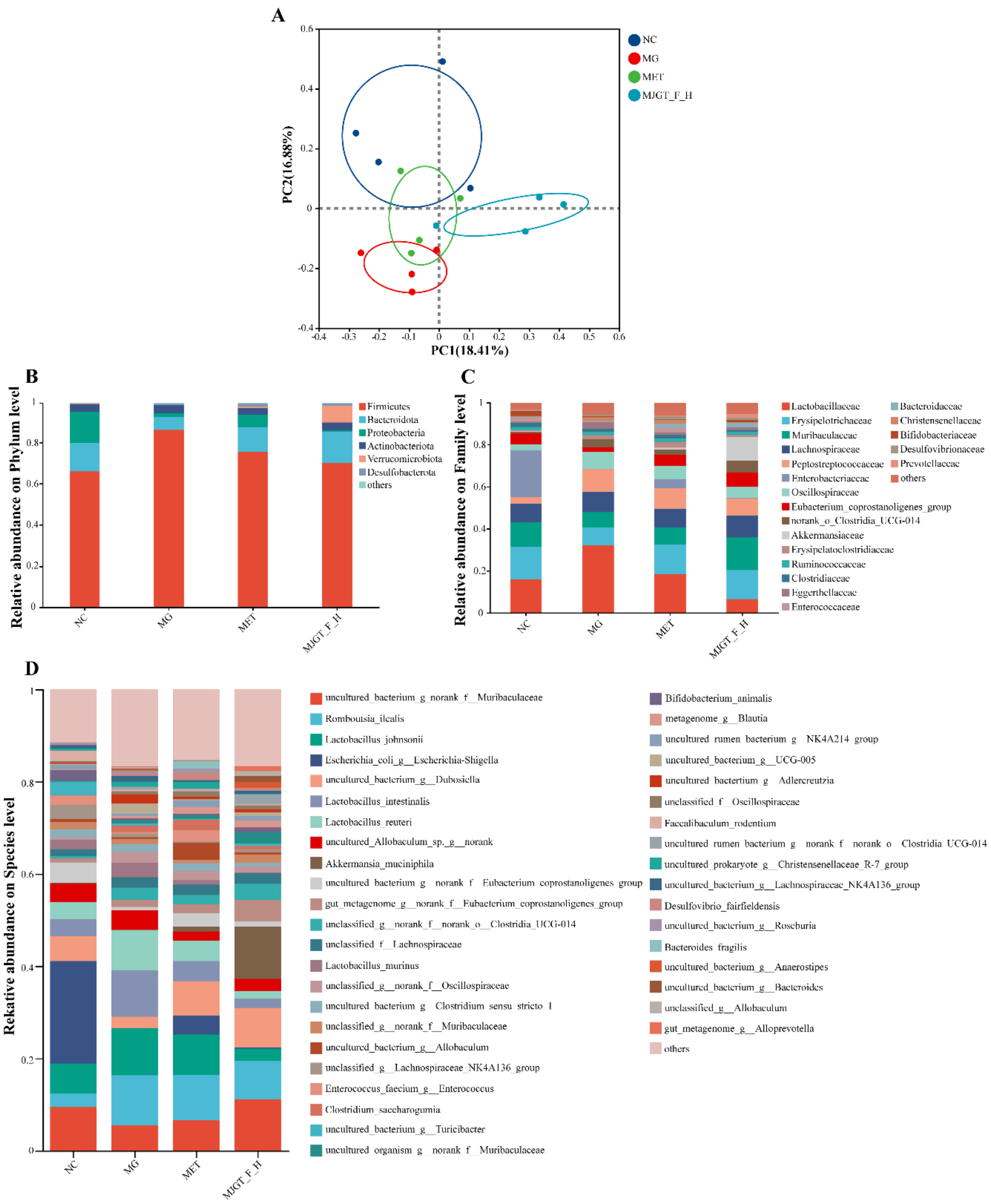

Figure S1. PCoA analysis of the OTU level of gut microbiota (A). The variations in gut microbiota composition are displayed the phylum (B), family (C) and species level (D), respectively. (*n*= 4 for each group). NC: Negative Control Group; MG: Model Group; MET: Metformin Hydrochloride Group; MJGT_F_H: the high-dose group of flavonoid extract from MJGT.
